# Identification and validation of platelet activation-related signatures in ulcerative colitis: a study based on machine learning and single-cell transcriptomic analysis

**DOI:** 10.3389/fimmu.2026.1855177

**Published:** 2026-06-19

**Authors:** Lu Zhao, Yang Liu, Shuqing Wang, Yujie Cai, Jing Zhi, Luqing Zhao, Shengsheng Zhang

**Affiliations:** 1 Digestive Disease Center, Beijing Hospital of Traditional Chinese Medicine, Capital Medical University, Beijing, China; 2 Department of Acupuncture, First Teaching Hospital of Tianjin University of Traditional Chinese Medicine, Tianjin, China; 3 National Clinical Research Center for Chinese Medicine Acupuncture and Moxibustion, Tianjin, China; 4 Beijing University of Chinese Medicine, Beijing, China

**Keywords:** bioinformatics analysis, immune dysregulation, machine learning, platelet activation, ulcerative colitis

## Abstract

**Background:**

Platelet activation (PA) acts as a molecular bridge connecting thrombosis and inflammation. This study aimed to identify key PA-related genes (PARGs) in ulcerative colitis (UC), and explore their transcriptional associations with immune–stromal dysregulation.

**Methods:**

Transcriptomic data of UC patients were obtained from the GEO database, and PARGs were retrieved from the MSigDB database. Differential expression analysis, WGCNA, LASSO, SVM-RFE, and random forest algorithms were applied to the GSE87466 dataset to identify key genes. Functional enrichment and immune infiltration analyses were performed to characterize their biological features. Additionally, single-cell RNA sequencing (scRNA-seq) analysis of the GSE214695 dataset was conducted to clarify their expression and localization. Findings were validated using independent GEO cohorts (GSE47908, GSE38713, and GSE36807) and qRT-PCR in a dextran sodium sulfate (DSS)-induced colitis mouse model.

**Results:**

We identified 22 PARGs in UC, which were associated with extracellular matrix (ECM) remodeling, platelet activation, and immune cell recruitment. Machine learning algorithms refined these to three key genes: SPARC, TIMP1, and SERPINA1. ROC analysis demonstrated robust diagnostic performance (AUC > 0.8) across the training and external validation cohorts. Crucially, single-cell analysis revealed that these genes were predominantly expressed in intestinal fibroblasts. Their expression levels strongly correlated with the infiltration of pathogenic immune cells (e.g., M1 macrophages, neutrophils). Additionally, an upstream regulatory network predicted transcription factors such as NFKB1 and SP1 as potential regulators. Finally, qRT-PCR confirmed the significant upregulation of these three genes in the DSS-induced colitis model.

**Conclusion:**

This study highlights the role of platelet activation in UC; identifies SPARC, TIMP1, and SERPINA1 as potential biomarkers; and provides important insights for the diagnosis and development of therapies for UC.

## 1 Introduction

Ulcerative colitis (UC) is a chronic and intractable inflammatory bowel disease characterized by continuous mucosal inflammation. Patients are typically distressed by bloody stools, abdominal pain, and diarrhea (1). Its etiology is complex and multifactorial, involving genetic susceptibility, epithelial barrier damage, dysregulated immune responses, and environmental influences. Contemporary surveillance revealed a striking annual prevalence of UC with 505/100,000 in Europe and 286/100,000 in North America, posing substantial socioeconomic burden (2). Currently, clinical practice increasingly relies on specific biomarkers (e.g., fecal calprotectin, anti-αvβ6 antibodies) for diagnosis and disease activity monitoring. However, these biomarkers primarily reflect general intestinal inflammation and fail to capture the progressive mucosal remodeling or microvascular changes. Moreover, despite therapeutic advancements from conventional immunosuppressants to targeted agents (JAK inhibitors and anti-integrin biologics), 10%–20% of patients suffer from primary non-response or secondary loss of efficacy, leaving them at high risk for disease relapse, irreversible tissue damage, and colitis-associated colorectal cancer (3–5). Emerging evidence suggests that UC refractoriness is largely driven by a dysregulated intestinal microenvironment. Due to the lack of effective biomarkers that reflect deep tissue pathological features, many patients miss the optimal intervention window, which exacerbates mucosal injury and accelerates disease progression (6, 7). Consequently, characterizing the UC intestinal microenvironment and identifying marker genes associated with disease progression are critical for improving disease management and achieving long-term remission.

Platelet activation is a key biological process that maintains vascular integrity and regulates immune responses, playing an important role in responding to endothelial injury and initiating tissue repair (8). This process occurs not only in peripheral blood but also serves as a critical link between hemostasis and inflammation in injured tissues (9). In UC, sustained disruption of the intestinal mucosal barrier drives aberrant platelet activation (10). This aberrant activation not only impairs intestinal microcirculation and promotes local microthrombus formation but, more importantly, enables activated platelets to infiltrate the injured mucosa and engage in complex crosstalk with immune and stromal cells (10, 11). Studies have demonstrated that this intercellular crosstalk is closely associated with the release of inflammatory factors and abnormal extracellular matrix (ECM) deposition, both of which are key drivers of tissue remodeling and fibrosis progression in UC (10). Therefore, elucidating the transcriptomic signatures of platelet activation in UC may facilitate the identification of candidate markers that reflect this complex immune–stromal microenvironment, thereby offering novel molecular insights to optimize disease intervention strategies.

In this study, we applied three machine learning algorithms, LASSO, random forest (RF), and support vector machine-recursive feature elimination (SVM-RFE), to identify three key candidate platelet activation-related genes (PARGs) in UC from multicohort transcriptomic data. Based on these three key genes, we systematically evaluated their predictive performance and expression levels in both training and validation cohorts. We then performed a multidimensional analysis of these candidate key genes, including single-gene set enrichment analysis (GSEA), immune infiltration assessment, upstream regulatory network construction (miRNA–TF–mRNA), and single-cell localization mapping in the intestinal microenvironment. Finally, we conducted preliminary experimental validation of the candidate key genes in the DSS-induced UC mouse model to verify our transcriptomic findings in an *in vivo* disease environment, thereby exploring their realistic expression patterns during active mucosal injury. Collectively, this study revealed the role of platelet activation in the intestinal microenvironment of UC from a transcriptomic perspective, providing new insights for assessing tissue remodeling risk and exploring therapeutic targets.

## 2 Materials and methods

### 2.1 Data acquisition and preprocessing

Transcriptomic expression matrices were obtained from the Gene Expression Omnibus (GEO) database (http://www.ncbi.nlm.nih.gov/geo/). Dataset selection criteria were as follows: 1) expression profiling performed by microarray or high-throughput sequencing, 2) samples limited to human intestinal mucosal tissue, and 3) inclusion of both UC patient and normal control (NC) samples. Based on these criteria, this study included four bulk transcriptomic datasets and one single-cell RNA sequencing (scRNA-seq) dataset. Specifically, GSE87466 (87 UC vs. 21 NC), based on the GPL13158 platform, was used as the training set. Three independent datasets based on the GPL570 platform—GSE47908 (45 UC vs. 15 NC), GSE38713 (30 UC vs. 13 NC), and GSE36807 (15 UC vs. 7NC)—served as external validation sets. The scRNA-seq dataset GSE214695 (6 UC vs. 6 NC) was used for single-cell analysis. Detailed information for each dataset is provided in **Table 1**. Platelet activation-related genes (PARGs) were obtained from the Molecular Signatures Database (https://www.gsea-msigdb.org/). The gene set designated REACTOME_PLATELET_ACTIVATION_SIGNALING_AND_AGGREGATION, which comprehensively covers the signaling cascade and aggregation processes of activated platelets, was downloaded to extract the target PARGs.

**Table 1:** Details of the UC datasets.

GEO number	Platform	Tissue	UC	Control	References	Group
GSE87466	GPL13158	Colonic mucosa	87	21	PMID: 29401083	Training cohort
GSE47908	GPL570	Colonic mucosa	45	15	PMID: 25358065	Validation cohort
GSE38713	GPL570	Colonic mucosa	30	13	PMID: 23135761	Validation cohort
GSE36807	GPL570	Colonic mucosa	15	7	PMID: 24155895	Validation cohort
GSE214695	GPL18573	Colonic mucosa	6	6	PMID: 37495570	ScRNA-seq

All raw data processing was performed using R software (version 4.5.1). After obtaining the expression matrices via the “GEO query” R package, we conducted log2 transformation to ensure data comparability. The “normalizeBetweenArrays” function from the “limma” R package was then applied for data normalization. Subsequently, microarray probes were precisely mapped to their corresponding gene symbols, and redundant probes were removed to obtain unique gene expression profiles. Because each external cohort was used for independent validation, batch correction across datasets was not performed in this study, thereby preserving the original data characteristics.

### 2.2 Identification of differentially expressed genes in UC

To identify differentially expressed genes (DEGs) between UC and normal control samples, we performed differential expression analysis on the training set (GSE87466) using the “limma” R package, with |logFC| >1 and adj.P-value <0.05. Heatmaps were generated using the “pheatmap” R package, and volcano plots were constructed using the “ggplot2” R package to visualize the results.

### 2.3 Weighted gene co-expression network analysis in UC

To further identify gene co-expression modules associated with UC, we performed weighted gene co-expression network analysis (WGCNA) using the “WGCNA” R package. First, the expression matrix was filtered by variance, retaining the top 50% most variable genes for subsequent analysis. Sample quality was then assessed by hierarchical clustering, and outliers were removed. During network construction, the soft threshold power of *β* was set to 16 to ensure a scale-free topology. Based on the adjacency matrix calculated under this threshold, the topological overlap matrix (TOM) and its corresponding dissimilarity matrix (1 − TOM) were derived to evaluate gene connectivity. Subsequently, a phylogenetic clustering tree was constructed to group genes with similar expression patterns into the same modules, with the minimum module size set to 30 and the module merging height cut at 0.25. To identify modules most relevant to UC, we calculated the module eigengenes (MEs) for each module, evaluated their Pearson correlation with UC, and selected the top 2 modules with the highest absolute correlation coefficients. Genes from these two modules were then intersected with DEGs to obtain UC-associated genes. Finally, these genes were subjected to a secondary intersection with PARGs. The results were visualized using Venn diagrams.

### 2.4 Functional enrichment analysis of PARGs in UC

GO and KEGG enrichment analyses of PARGs in UC were performed by the “clusterProfiler” R package to explore their biological functions. Metascape (https://metascape.org) was used to further consolidate the functional information and build potential protein–protein networks, with the parameters set as minimum overlap = 3 and minimum enrichment = 1.5.

### 2.5 Identification of key PARGs in UC by machine learning

To identify key PARGs in UC, three machine learning algorithms were applied to the training cohort (GSE87466). The least absolute shrinkage and selection operator (LASSO) algorithm penalizes high-dimensional data through L1 regularization, effectively reducing overfitting risk. We employed 10-fold cross-validation to determine the optimal penalty parameter (*λ*) and extracted genes with non-zero coefficients at lambda.min. The random forest (RF) model was constructed using the “randomForest” R package. As an ensemble learning algorithm, RF improves prediction accuracy and robustness by building a large number of decision trees. Variable importance was ranked based on the mean decrease in Gini impurity, and the top 10 genes were extracted as candidate features. The SVM-RFE algorithm was implemented using the “e1071” R package. SVM-RFE is a margin-maximization-based classifier that iteratively removes the least contributory features to identify the optimal gene combination. We used 5-fold cross-validation to evaluate the classification performance across feature subsets, and selected the feature set with the lowest classification error rate. Finally, a Venn diagram was used to identify overlapping genes recognized by all three algorithms, which were then subjected to subsequent analysis and external validation.

### 2.6 Evaluation and validation of key genes

To evaluate the diagnostic performance of the identified key genes in UC, receiver operating characteristic (ROC) curves were generated using the “pROC” R package, and the area under the curve (AUC) was calculated to quantify classification performance. An AUC value closer to 1 indicates better diagnostic performance, and in this study, an AUC exceeding 0.7 was considered highly diagnostic for UC. This evaluation was performed in the training set (GSE87466) and three independent external validation sets (GSE36807, GSE38713, and GSE47908) to ensure robustness of the results. Additionally, volcano plots were used to visualize gene expression differences, and violin plots were used to visualize and statistically compare the relative expression levels of each key gene between the UC and normal control groups.

### 2.7 Single-gene GSEA of key genes

To investigate the potential biological functions and related signaling pathways of the key genes in UC, single-gene set enrichment analysis (GSEA) was performed using the “clusterProfiler” R package. For each key gene, UC samples were stratified into high- and low-expression groups based on the median expression level. The expression difference (fold change) of all genes between the two groups was then calculated and ranked in descending order. The KEGG canonical pathway gene set (c2.cp.kegg.Hs.symbols.gmt) was obtained from the MSigDB database as the reference. Finally, the top 5 most significantly enriched pathways (both positively and negatively correlated) in the high- versus low-expression groups were extracted and visualized using the “enrichplot” R package.

### 2.8 Immune infiltration analysis

The CIBERSORT deconvolution algorithm was used to quantify the differences in the immune microenvironment between UC and normal control samples. CIBERSORT is a computational method based on linear support vector regression (SVR) that transforms a normalized gene expression matrix from mixed tissues into relative proportions of 22 infiltrating immune cells. The LM22 leukocyte signature gene matrix was used as the reference set, with 1,000 permutations to improve prediction accuracy. As the training set (GSE87466) is microarray data, quantile normalization (QN = TRUE) was enabled to correct for systematic biases. To ensure statistical reliability, only samples with a CIBERSORT output *P*-value <0.05 were selected for subsequent analyses. Stacked bar plots and boxplots were generated to visualize immune cell composition differences between the UC and normal control groups. Spearman correlation analysis was further performed to evaluate associations between the identified key genes and immune cell proportions.

### 2.9 Single-cell analysis

To explore the expression patterns of the key genes in the intestinal microenvironment at the single-cell level, the scRNA-seq dataset (GSE214695) was analyzed using the “Seurat” R package. The following quality control (QC) criteria were applied: 1) genes expressed in at least three cells were retained; 2) cells with fewer than 200 or more than 5,000 genes, or with total transcript counts <200 or >30,000, were excluded; and 3) cells with mitochondrial gene >30% or hemoglobin gene >5% were removed. After stringent QC, the remaining high-quality cells were normalized using the “LogNormalize” method, and 2,000 highly variable genes were identified using the “vst” method for data scaling and principal component analysis (PCA). Given that the dataset comprised multiple independent samples, the “harmony” R package was introduced to remove batch effects. Unsupervised clustering was then performed based on the batch-corrected principal components, and the clustering results were visualized using the uniform manifold approximation and projection (UMAP) algorithm. Finally, by referencing the CellMarker 2.0 database and previously reported marker genes in the literature, each cell cluster was manually annotated, allowing for the identification of major cell subsets.

### 2.10 miRNA–TF–mRNA network construction

To investigate upstream regulators of key genes, the miRTarBase (https://mirtarbase.cuhk.edu.cn) and TRRUST (https://www.grnpedia.org/trrust) databases were utilized to predict potential microRNAs (miRNAs) and transcription factors (TFs). The predicted upstream regulators were then integrated with the key genes to construct a miRNA–TF–mRNA network using Cytoscape.

### 2.11 Animal experiments

Eight-week-old male C57BL/6 mice were obtained from Speafu Biotechnology Co., Ltd. (Beijing, China). After 1 week of acclimatization under controlled conditions (temperature: 20 °C−24 °C, humidity: 45%–65%, 12-h light/dark cycle), mice were randomly assigned to a normal control group (*n* = 6) or a model group (*n* = 6). Acute colitis was induced in the model group by administering 3% dextran sodium sulfate (DSS, MeilunBio, 9011-18-1) solution for 7 days, whereas the control group received equal amounts of distilled water. During the 7-day period, body weight, stool consistency, and fecal occult blood were recorded daily for each mouse by two researchers blinded to group allocation. The disease activity index (DAI) scoring was performed according to the criteria described by Cooper et al. (12), specifically weight loss (0 = none, 1 = 1%–5%, 2 = 5%–10%, 3 = 10%–15%, 4 ≥ 15%), stool consistency (0 = normal, 2 = loose stool, 4 = diarrhea), and fecal occult blood (0 = none, 2 = fecal occult blood, 4 = severe bloody stool and perianal blood). Model success was defined as significant weight loss, elevated DAI, and rectal bleeding. On day 8, mice were euthanized, and the entire colon was immediately excised for length measurement and photography. Colonic tissues were collected; one portion was fixed in 4% paraformaldehyde for H&E staining to assess histopathological injury, and another portion was flash-frozen in liquid nitrogen and stored at −80°C for subsequent RNA extraction. All animal procedures were approved by the Ethical Review Committee for Animal Experiments of Beijing Hospital of Traditional Chinese Medicine, Capital Medical University (approval no. BJTCM-M-2025-06-07).

### 2.12 Quantitative real-time PCR

Total RNA was isolated from colon tissues using the RNA Simple Kit (TIANGEN, DP419, Beijing, China). Reverse transcription and amplification were conducted using the FastKing RT kit (TIANGEN, KR118-02) and Taq Pro Universal SYBR qPCR Master Mix (Vazyme, Q712-02, Nanjing, China) kit, respectively. GAPDH was used as the internal control, and 2^−ΔΔCt^ was used to show the relative expression of genes. The primer sequences used for quantitative real-time PCR (qRT-PCR) are provided in **Table 2**. Graphical data were plotted using GraphPad Prism.

**Table 2:** Primer sequences for qRT-PCR.

Gene	Base pairs	Forward primers (5′–3′)	Reverse primers (5′–3′)
SPARC	276	CAGAAGCTGCGTGTGAAGAAGAT	CAGGGCAATGTACTTGTCGTTGT
TIMP1	122	GCAACTCGGACCTGGTCATAAG	TCCCACAGCCTTGAATCCTTT
SERPINA1	132	CTCCTCCAAACCCTCAACAGA	GACTTCTGCCTGATAATGGTTCTT

### 2.13 Statistical analysis

Bioinformatics analyses and data visualizations were performed using R software (version 4.5.1). For differential expression analysis of transcriptomic data, the Benjamini–Hochberg (BH) method was applied to adjust *P*-values for multiple testing, controlling the false discovery rate (FDR). To evaluate the diagnostic performance of the key genes, the AUC and its 95% confidence interval (95% CI) were calculated. Correlations between key genes and immune cells were assessed by the Pearson method. All experimental data were expressed as mean ± standard deviation (SD) and were statistically analyzed/visualized by GraphPad Prism (version 9.5.0). Normality of data distribution was assessed using the Shapiro–Wilk test. Differences between the two groups were compared using Student’s *t*-test (for normally distributed data) or Kruskal–Wallis test (for non-normally distributed data). A *P*-value <0.05 was considered statistically significant.

## 3 Results

### 3.1 Integration of differential expression analysis and WGCNA for UC-associated gene selection

**Figure 1:**
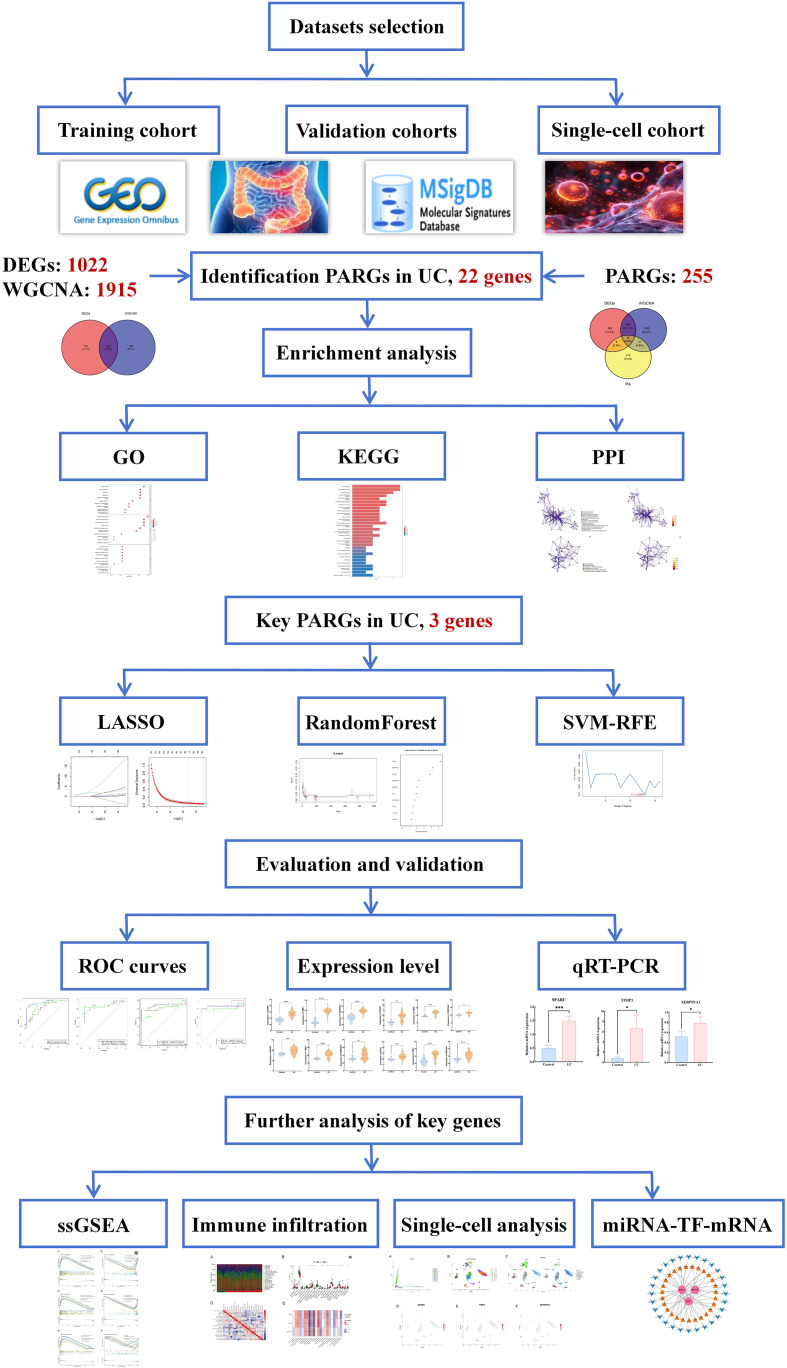
The workflow of this study.

**Figure 2:**
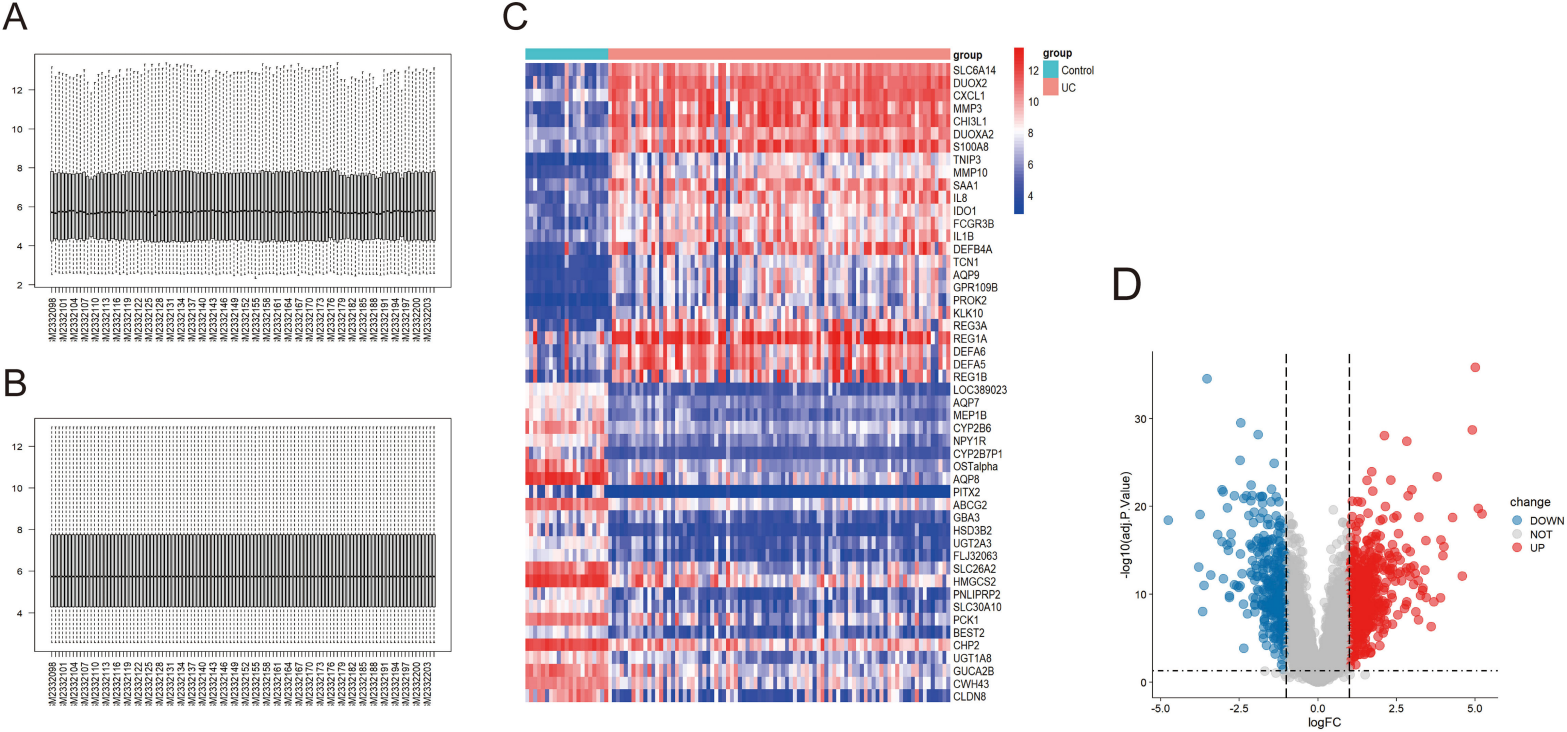
Data pre-processing and identification of differentially expressed genes (DEGs). **(A)** Raw data of GSE87466 before normalization. **(B)** Data of GSE87466 after normalization. **(C)** Heatmap of DEGs in GSE87466. **(D)** Volcano plot of DEGs in GSE87466.

**Figure 3:**
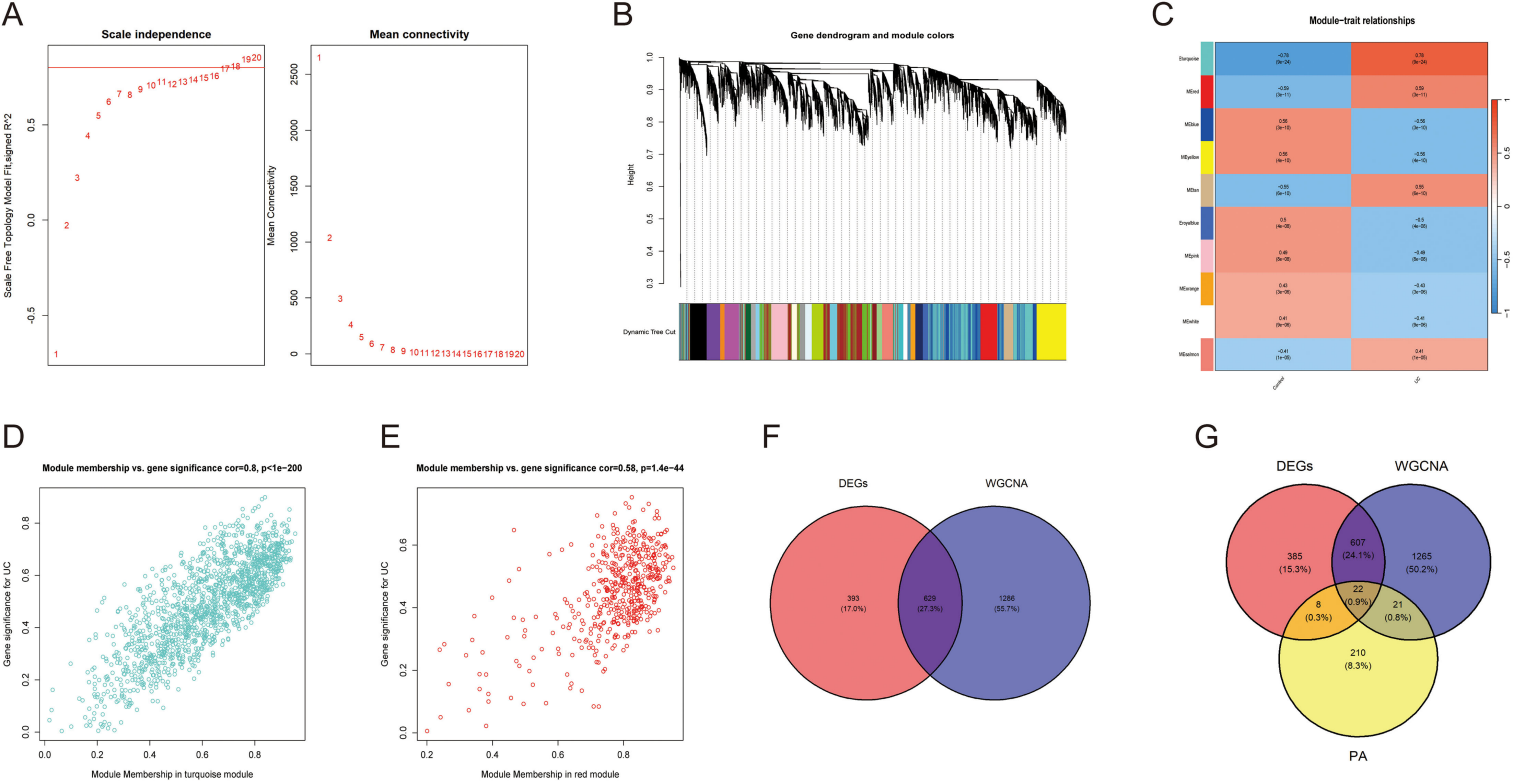
The results of weighted gene co-expression network analysis (WGCNA) analysis. **(A)** Selection of soft threshold. **(B)** Dendrogram of the clustering tree of co-expression modules. **(C)** Heatmap of UC module–trait relationships. **(D)** Genes of the turquoise module. **(E)** Genes of the red module. **(F)** Venn diagram of overlapping DEGs and WGCNA module genes. **(G)** Venn diagram of overlapping UC-associated genes and platelet activation-related genes (PARGs).

The overall analytical workflow of this study is illustrated in **Figure 1**. The data of GSE87466 were normalized (**Figures 2A, B**). A total of 1,022 DEGs (651 upregulated, 371 downregulated) were identified and visualized using a heatmap (**Figure 2C**) and a volcano plot (**Figure 2D**). WGCNA was performed with a soft threshold of 16 to achieve a scale-free distribution and to confirm the smoothness of connectivity tendency (**Figure 3A**). After merging highly correlated modules, 10 modules were identified for further analysis, and the clustering tree is shown in **Figure 3B**. Module–trait relationships are illustrated in **Figure 3C**. The turquoise (*R* = 0.78, *P* < 0.05, 1,434 genes) and red modules (*R* = 0.59, *P* < 0.05, 481 genes) were significantly correlated with UC (**Figures 3D, E**). Finally, the intersection of DEGs with the turquoise and red WGCNA modules yielded 629 UC-associated genes (**Figure 3F**).

### 3.2 Identification of PARGs in UC and functional enrichment analysis

**Figure 4:**
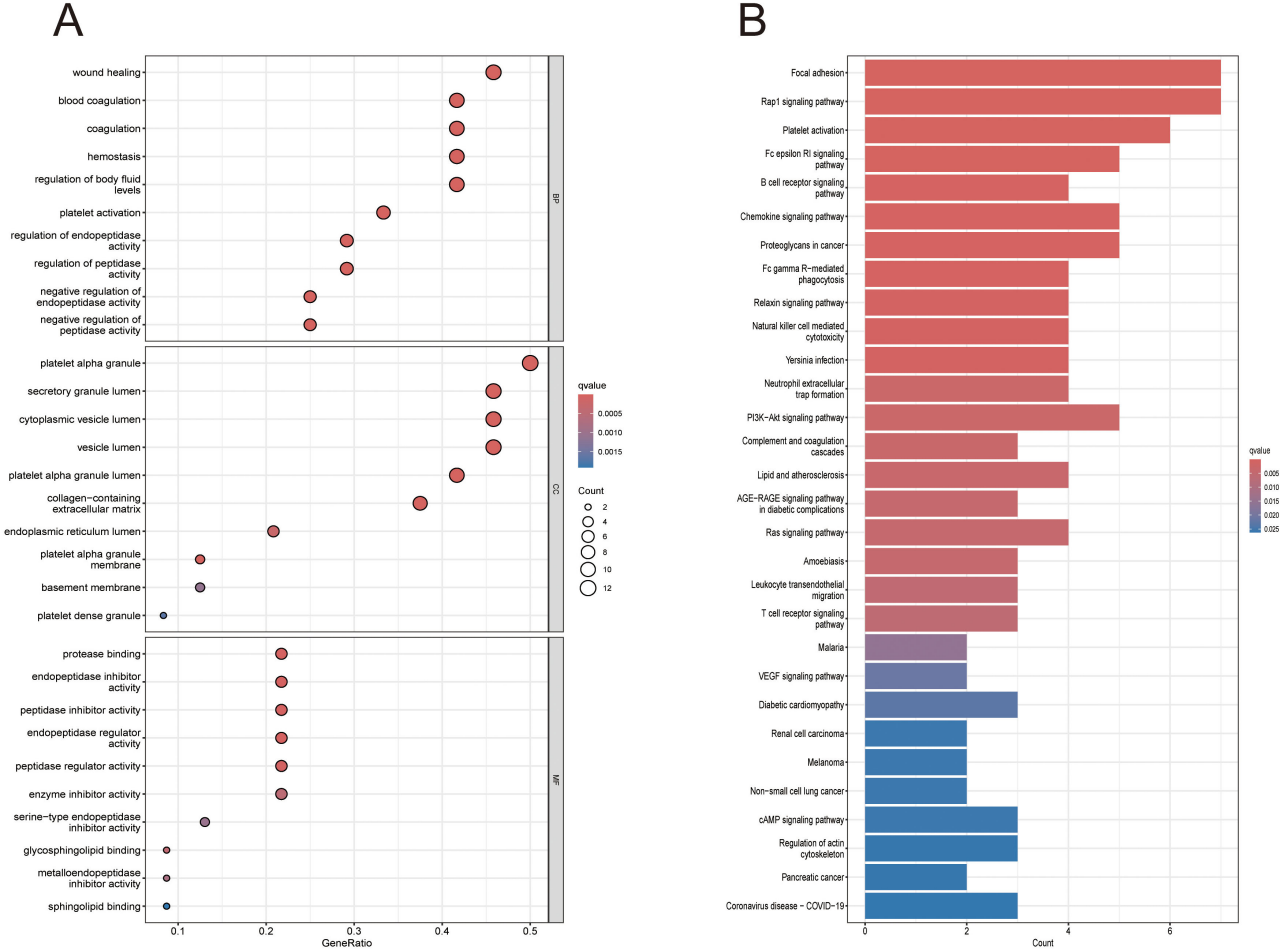
Functional enrichment analysis of PARGs in UC. **(A)** Bubble plot of GO analysis for PARGs in UC. **(B)** Bar plot of KEGG analysis for PARGs in UC.

**Figure 5:**
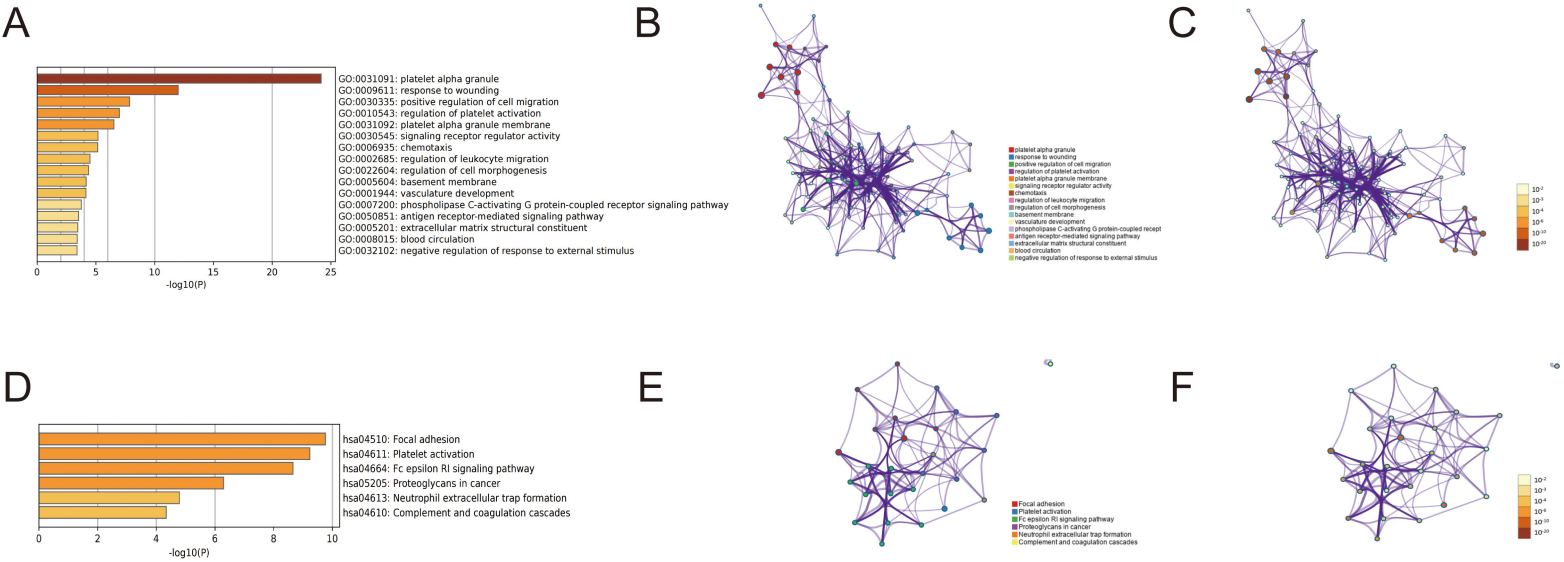
Integrative analysis of PARGs in UC using Metascape. **(A)** Bar plot of GO for PARGs in UC. **(B)** Network of GO colored by cluster ID. **(C)** Network of GO colored by cluster *P*-value. **(D)** Bar plot of KEGG for PARGs in UC. **(E)** Network of KEGG colored by cluster ID. **(F)** Network of KEGG colored by cluster *P*-value.

From MSigDB, 255 PARGs were retrieved and intersected with UC-associated genes, resulting in 22 overlapping genes that were identified as PARGs in UC (**Table 3**; **Figure 3G**). To explore the biological significance of these candidate genes in UC, a comprehensive functional enrichment analysis was performed. In the Biological Process (BP) and Cellular Component (CC) categories of GO analysis, these genes were highly enriched in blood coagulation, hemostasis, platelet alpha granules, and secretory granule lumen, suggesting a close association with platelet degranulation events. Molecular Function (MF) analysis revealed significant enrichment in protease binding and endopeptidase inhibitor activities, which typically regulate the proteolytic system (**Figure 4A**). KEGG analysis extended these findings, showing that the genes were enriched not only in the “platelet activation” pathway but also significantly enriched in “focal adhesion,” “chemokine signaling pathway,” and “leukocyte transendothelial migration” (**Figure 4B**). To further explore protein–protein interactions and gain broader pathway insights, we conducted an integrative analysis using the Metascape platform. These results reinforced the roles of these genes in platelet activation, immune cell recruitment, and extracellular matrix interaction (**Figure 5**). Additionally, Metascape highlighted other immune-relevant processes, including neutrophil extracellular trap (NET) formation and complement activation. Collectively, integration of GO, KEGG, and Metascape results revealed a highly coordinated biological process during UC progression, spanning from platelet-specific granule release to local tissue adhesive interactions and subsequent leukocyte transendothelial migration. This suggests that early platelet activation events may closely synergize with the subsequent recruitment of inflammatory and immune cells in the intestinal microenvironment.

**Table 3:** The 22 overlapping PARGs in UC.

Serial number	Gene symbol	Description	Function
1	TIMP1	Tissue inhibitor of metalloproteinase 1	Inhibits matrix metalloproteinases; regulates cell proliferation, apoptosis, and ECM remodeling
2	SERPINA1	Serpin family A member 1 (alpha-1 antitrypsin)	Serine protease inhibitor; the primary target is neutrophil elastase; modulates inflammatory response
3	LYN	LYN proto-oncogene, Src family tyrosine kinase	Non-receptor tyrosine kinase involved in immune cell signaling, integrin regulation, and hematopoietic development
4	SPARC	Secreted protein acidic and cysteine-rich	Extracellular matrix glycoprotein mediating cell–matrix interactions, tissue repair, and anti-adhesion
5	PIK3R3	Phosphoinositide-3-kinase regulatory subunit 3	Regulatory subunit of the PI3K complex; links growth factor receptors to downstream signaling cascades
6	CALU	Calumenin	EF-hand calcium-binding protein; regulates calcium homeostasis, ER function, and inflammation
7	SERPING1	Serpin family G member 1	Inhibits complement C1r and C1s; critical regulator of classical and lectin complement pathways
8	SERPINA3	Serpin family A member 3	Serine protease inhibitor; targets chymotrypsin-like proteases and modulates inflammatory states
9	GNA15	G protein subunit alpha 15	Mediates GPCR signaling; activates phospholipase C-β; involved in immune and inflammatory responses
10	LCP2	Lymphocyte cytosolic protein 2	Adapter protein in T-cell receptor signaling; essential for T-cell activation and integrin function
11	VEGFC	Vascular endothelial growth factor C	Key lymphangiogenic factor; promotes lymphatic endothelial cell proliferation and migration via VEGFR-2/3
12	PDPN	Podoplanin	Transmembrane glycoprotein; regulates cell migration, invasion, and lymphatic vessel formation
13	TIMP3	Tissue inhibitor of metalloproteinase 3	Inhibits MMPs and ADAMs; involved in ECM remodeling, apoptosis, and tissue homeostasis
14	HGF	Hepatocyte growth factor	Pleiotropic growth factor; activates c-Met signaling, promoting mitogenesis, motility, and morphogenesis
15	PF4	Platelet factor 4 (CXCL4)	Platelet-derived CXC chemokine; neutralizes heparin, inhibits angiogenesis, and modulates immune responses
16	P2RY1	Purinergic receptor P2Y1	ADP-activated GPCR; mediates platelet aggregation and smooth muscle contraction via PLC activation
17	VWF	Von Willebrand factor	Multimeric glycoprotein; mediates platelet adhesion to subendothelium and stabilizes coagulation factor VIII
18	VAV3	Vav guanine nucleotide exchange factor 3	GEF for Rho GTPases; regulates cytoskeletal remodeling, integrin signaling, and lymphocyte activation
19	RAC2	Rac family small GTPase 2	Rho GTPase; regulates NADPH oxidase-dependent ROS production, cell migration, and phagocytosis
20	PCDH7	Protocadherin 7	Calcium-dependent cell-cell adhesion molecule; involved in neural development and synapse formation
21	PLEK	Pleckstrin	PKC substrate; expressed in hematopoietic cells; regulates cytoskeletal organization and membrane trafficking
22	ISLR	Immunoglobulin superfamily containing leucine-rich repeat	Extracellular matrix protein; modulates mesenchymal stem cell differentiation, adhesion, and migration

### 3.3 Identification of key PARGs in UC by three machine learning algorithms

**Figure 6:**
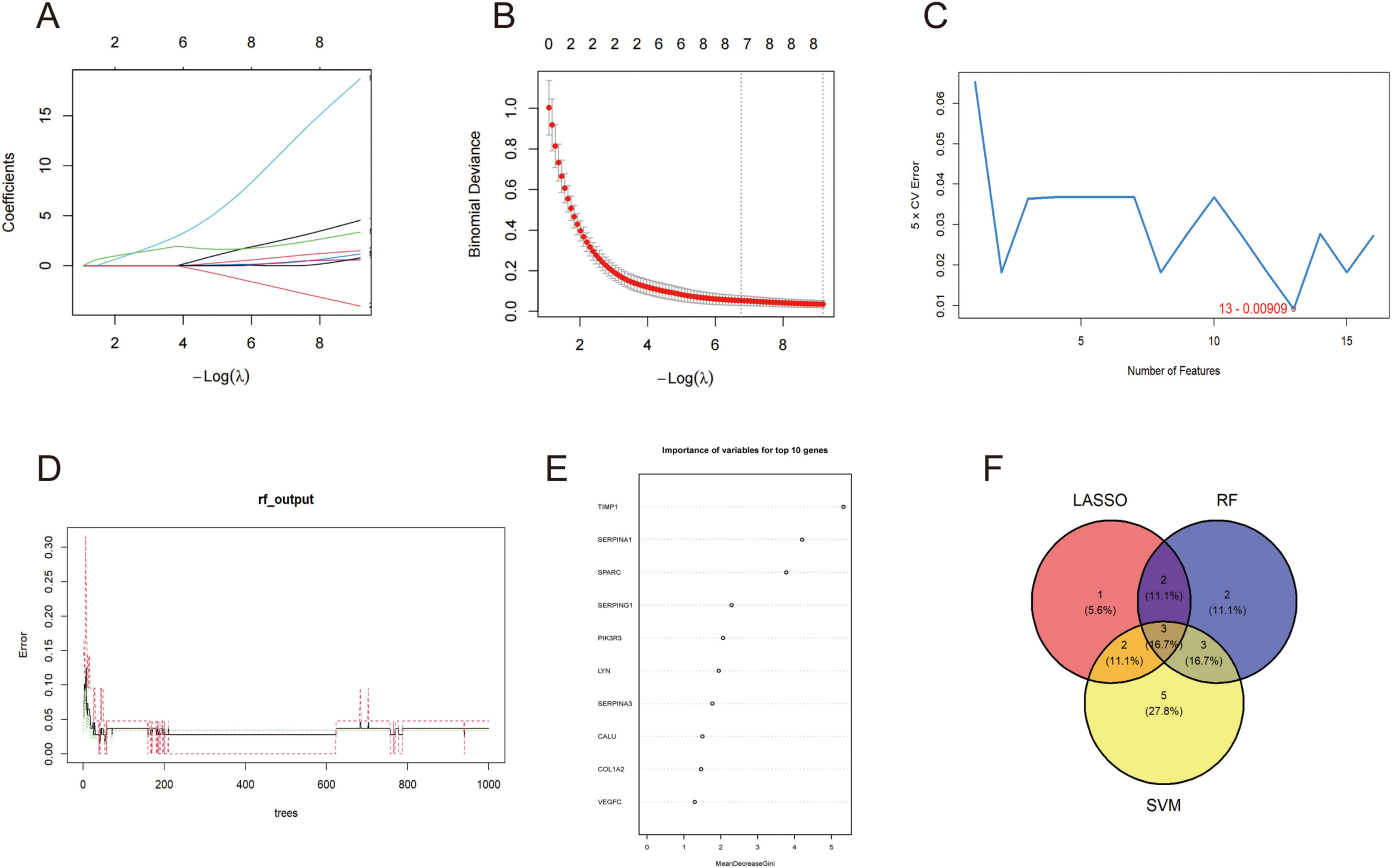
Identification of key PARGs in UC. **(A)** Cross-validation to select the best lambda in the least absolute shrinkage and selection operator (LASSO) regression analysis. **(B)** LASSO coefficient profiles of the key genes. **(C)** Support vector machine-recursive feature elimination (SVM-RFE) analysis identified 13 genes with an error of 0.009. **(D)** Random forest (RF) error rate confidence intervals. **(E)** RF showed the top 10 important genes. **(F)** Venn diagram of the three key genes shared by the machine learning algorithms.

Key PARGs in UC were selected using LASSO, SVM-RFE, and RF. LASSO regression resulted in eight genes (**Figures 6A, B**), SVM-RFE identified 13 genes (**Figure 6C**), and the RF model showed the top 10 genes (**Figures 6D, E**). The three overlapping key genes (SPARC, TIMP1, and SERPINA1) were then obtained by taking the intersection of the three algorithms and are shown in **Figure 6F**.

### 3.4 Evaluation and validation of key gene expression and diagnostic performance

**Figure 7:**
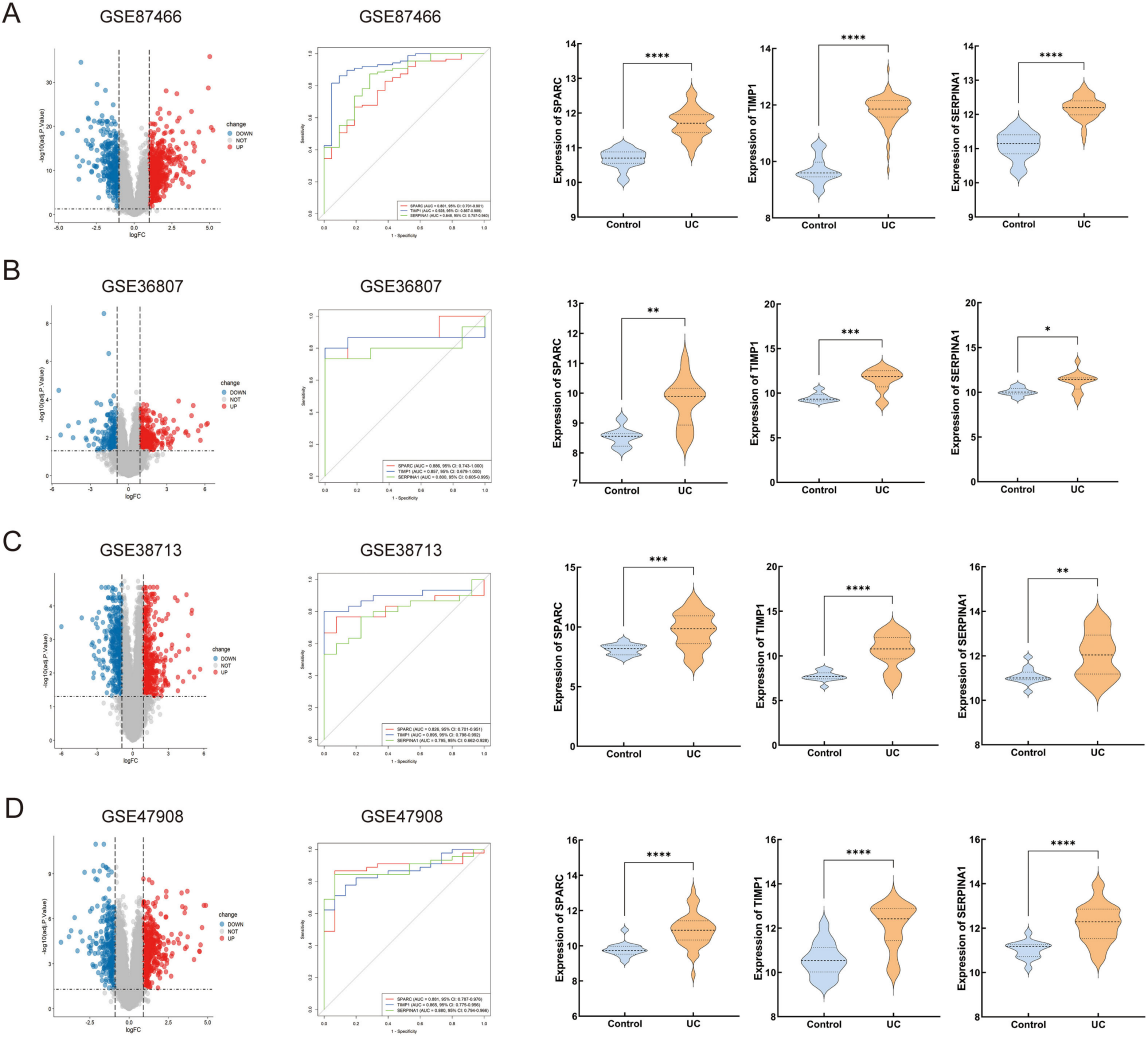
Evaluation and validation of key genes in UC. **(A)** The volcano plot showed DEGs of GSE87466, the ROC curve showed the diagnostic values of key genes in GSE87466, and the violin plot showed the expression of key genes in GSE87466. **(B)** The volcano plot showed DEGs of GSE36807, the ROC curve showed the diagnostic values of key genes in GSE36807, and the violin plot showed the expression of key genes in GSE36807. **(C)** The volcano plot showed DEGs of GSE38713, the ROC curve showed the diagnostic values of key genes in GSE38713, and the violin plot showed the expression of key genes in GSE38713. **(D)** The volcano plot showed DEGs of GSE47908, the ROC curve showed the diagnostic values of key genes in GSE47908, and the violin plot showed the expression of key genes in GSE47908. *P < 0.05, **P < 0.01, ***P < 0.001, ****P < 0.0001 vs. the control group.

To evaluate the expression and diagnostic potential of the key genes, we generated box plots and ROC curves and calculated AUC values using the GSE87466 dataset, followed by validation in three independent datasets (GSE47908, GSE38713, and GSE36807). The results showed that all three key genes were highly expressed across all four datasets. In the training set (GSE87466), the AUC values for SPARC, TIMP1, and SERPINA1 were 0.801 (95% CI: 0.701–0.901), 0.928 (95% CI: 0.867–0.989), and 0.848 (95% CI: 0.757–0.940), respectively (**Figure 7A**). In the validation sets, the AUC values were as follows: GSE36807 (**Figure 7B**): 0.886 (95% CI: 0.743–1.000), 0.857 (95% CI: 0.679–1.000), and 0.800 (95% CI: 0.605–0.995); GSE38713 (**Figure 7C**): 0.826 (95% CI: 0.701–0.951), 0.895 (95% CI: 0.798–0.992), and 0.795 (95% CI: 0.662–0.928); and GSE47908 (**Figure 7D**): 0.881 (95% CI: 0.787–0.976), 0.865 (95% CI: 0.775–0.956), and 0.880 (95% CI: 0.794–0.966), respectively. These results indicated that the three key genes have high predictive value for UC, supporting their potential utility in clinical diagnosis.

### 3.5 Single-gene GSEA analysis

**Figure 8:**
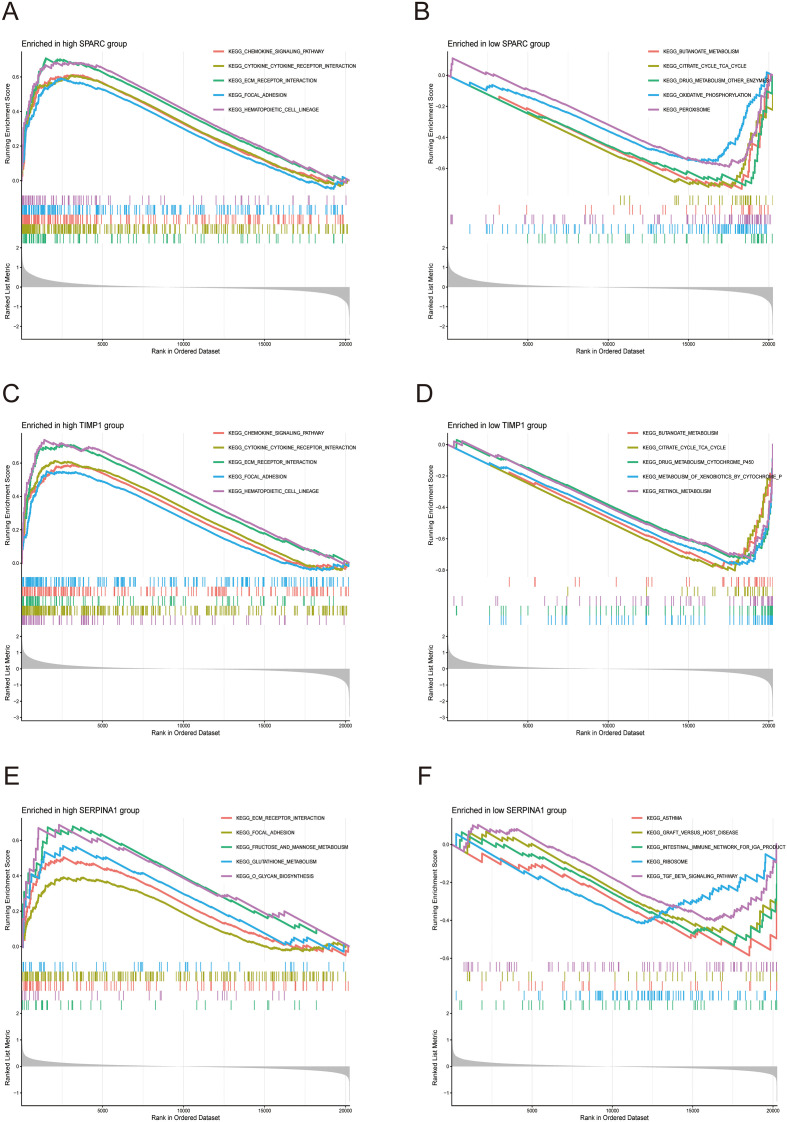
Single gene GSEA analysis on the key genes in UC. **(A)** Enrichment in the high SPARC group. **(B)** Enrichment in the low SPARC group. **(C)** Enrichment in high TIMP1 group. **(D)** Enrichment in the low TIMP1 group. **(E)** Enrichment in the high SERPINA1 group. **(F)** Enrichment in the low SERPINA1 group.

To further explore downstream signaling cascades potentially activated by the key genes, single-gene GSEA was performed. As shown in **Figure 8**, high-expression groups of SPARC, TIMP1, and SERPINA1 were significantly enriched in matrix remodeling-related pathways (e.g., “ECM receptor interaction” and “focal adhesion”) as well as pro-inflammatory pathways (e.g., “chemokine signaling pathway” and “cytokine–cytokine receptor interaction”). These results are highly consistent with the functional enrichment findings described above: upregulation of these genes correlates with extracellular matrix remodeling and immune cell recruitment. In contrast, low expression of these key genes was associated with metabolic homeostasis pathways, including “oxidative phosphorylation,” the “TCA cycle,” and “butyrate metabolism.” This indicated that low-expression levels of SPARC, TIMP1, and SERPINA1 may contribute to a healthy and metabolically stable colonic microenvironment. Collectively, the GSEA results demonstrated that aberrant expression of these key genes was strongly correlated with pathological features of the UC microenvironment. Their overexpression coincides with an aggressive “ECM remodeling–immune activation” state, whereas low expression aligns with mucosal metabolic homeostasis. These findings suggested that these genes may be potential participants in the pathological transition of UC.

### 3.6 Immune infiltration analysis

**Figure 9:**
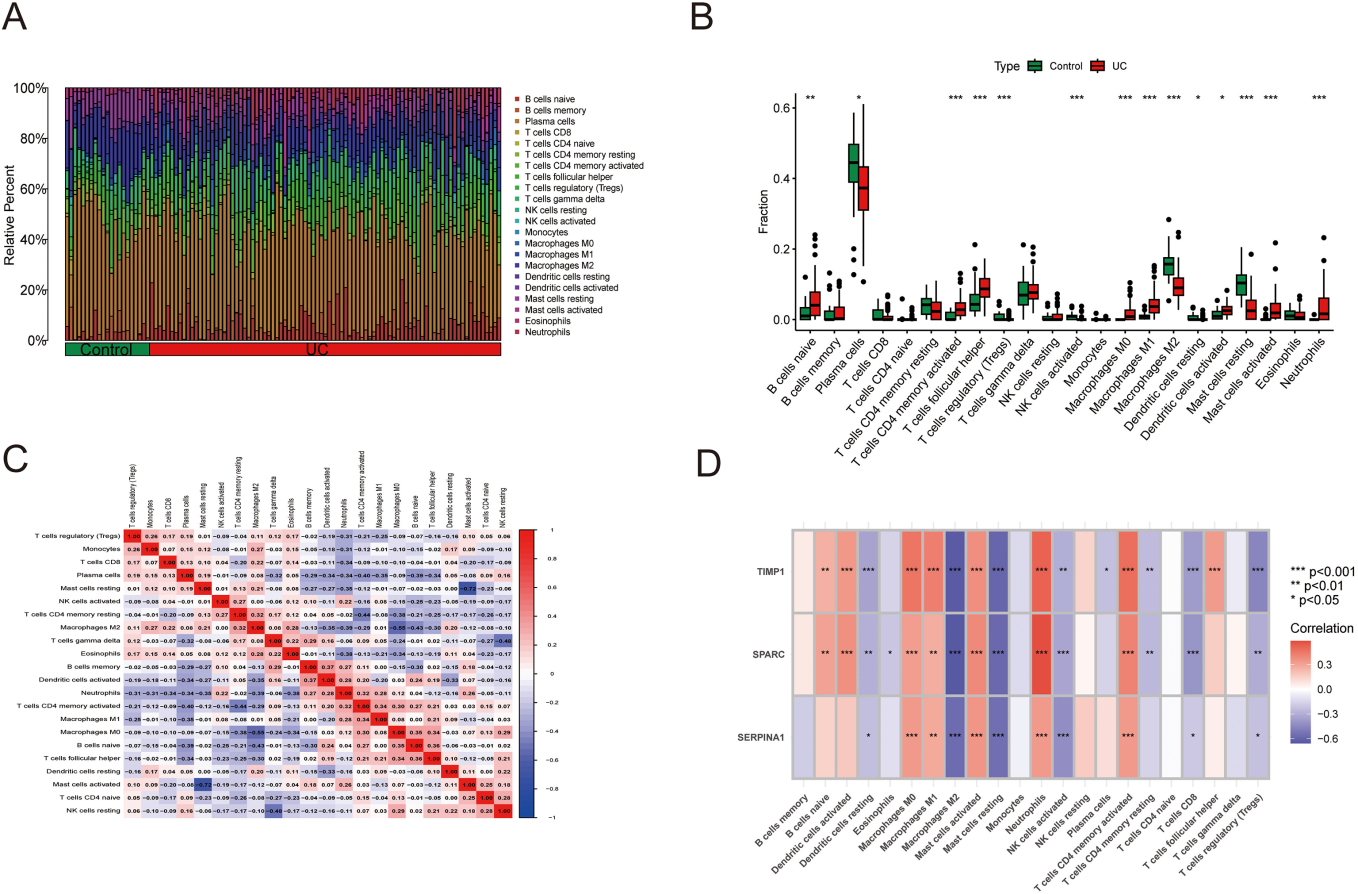
Immune infiltration in UC. **(A)** Heatmap of immune cell proportions in control and UC. **(B)** Box plot showed differences of immune cells between the control and UC. **(C)** Heatmap of immune cell correlation in UC. **(D)** Correlation analysis of key genes with immune cells.

CIBERSORT analysis revealed significant changes in the immune microenvironment of UC. As shown in **Figures 9A, B**, compared with healthy controls, the proportions of pro-inflammatory cell populations were significantly increased in UC, including naive B cells, activated memory CD4^+^ T cells, follicular helper T (Tfh) cells, M1 macrophages, activated mast cells, and neutrophils. In contrast, immunosuppressive or regulatory cell populations, such as regulatory T cells (Tregs), activated natural killer (NK) cells, M2 macrophages, and resting mast cells, were significantly reduced. Correlation analysis revealed a highly coordinated pattern of immune cell infiltration (**Figure 9C**). For example, M1 macrophages exhibited a positive correlation with activated memory CD4^+^ T cells (*r* = 0.34), whereas naive B cells showed a negative correlation with M2 macrophages (*r* = −0.43). Notably, the expression levels of the three key genes were strongly positively correlated with the abundance of pro-inflammatory cells (e.g., neutrophils, M1 macrophages, activated dendritic cells) and negatively correlated with immunosuppressive cells (e.g., M2 macrophages and Tregs) (**Figure 9D**). These robust correlation findings are highly consistent with the biological processes described above, suggesting a synergistic interaction between tissue matrix remodeling and immune responses during UC progression.

### 3.7 Single-cell analysis

**Figure 10:**
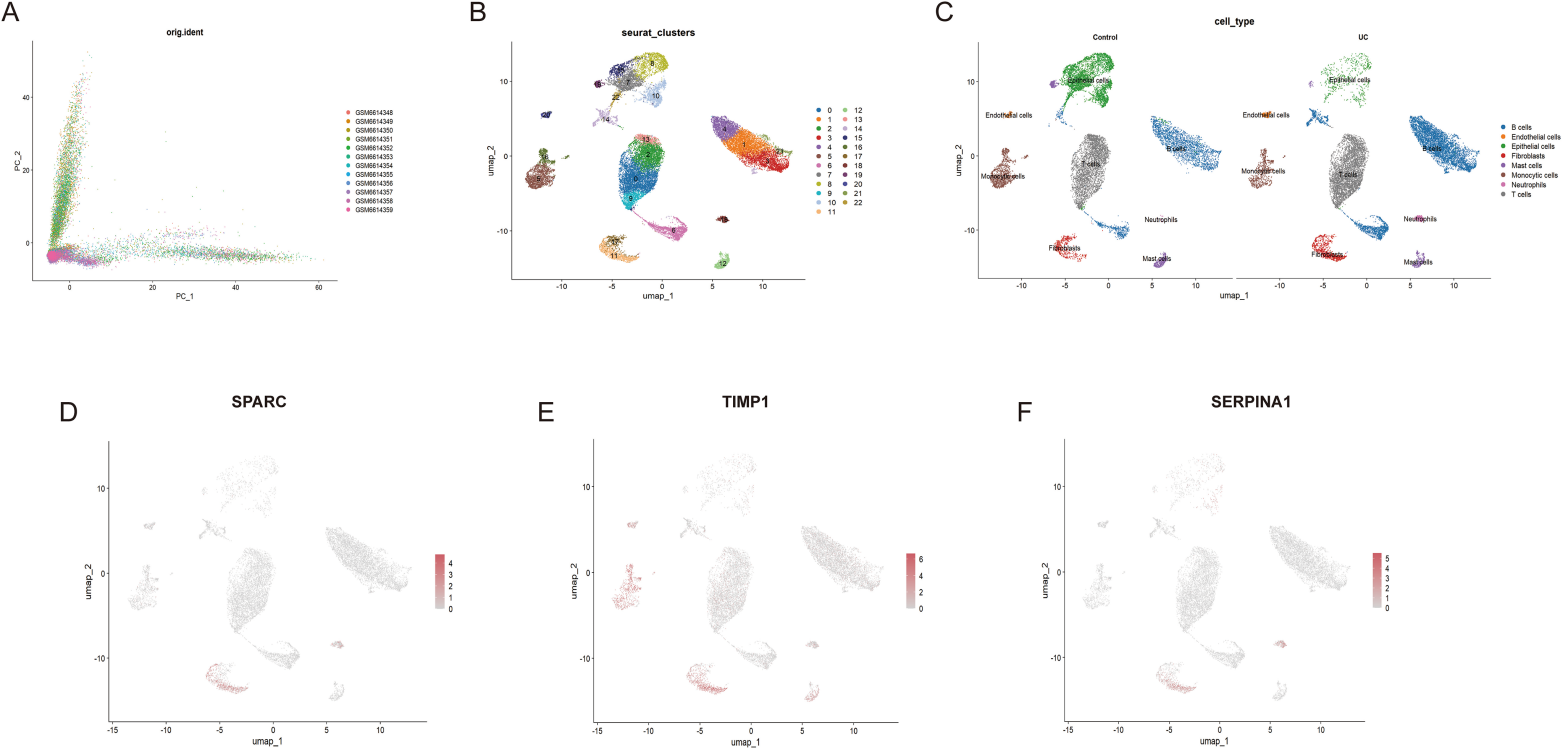
Single-cell distribution of key genes in GSE214695. **(A)** Scatter plot of the principal component. **(B)** The t-SNE plot showed major classifications involved in UC. **(C)** The t-SNE plot showed the major cell types in UC. **(D)** Distribution of SPARC across cell types in UC. **(E)** Distribution of TIMP1 across cell types in UC. **(F)** Distribution of SERPINA1 across cell types in UC.

The single-cell RNA-seq dataset GSE214695 (6 UC vs. 6 NC) was used to characterize the cellular distribution of the key genes. The elbow plot showed the variance explained by each principal component (**Figure 10A**). The top principal components were selected for dimensionality reduction and classification, yielding 22 cell clusters (**Figure 10B**). Compared with healthy controls, UC patients exhibited an increase in immune cells and a decrease in intestinal epithelial cells (**Figure 10C**). Genetic correlation analysis revealed that all three key genes were predominantly distributed in fibroblasts (**Figures 10D–F**).

### 3.8 Construction and analysis of the miRNA–TF–mRNA network

**Figure 11:**
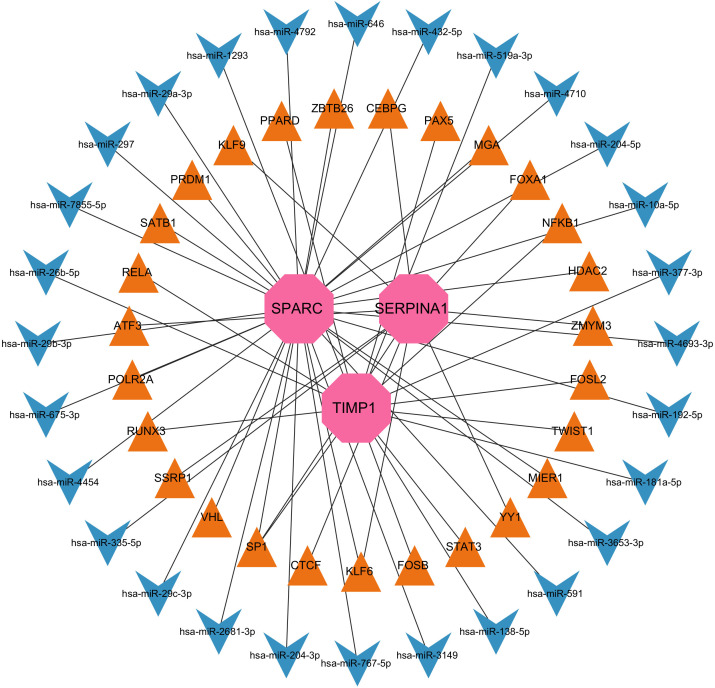
miRNA–TF–mRNA regulatory network.

To explore potential upstream regulators of the key genes, a miRNA–TF–mRNA interaction network was constructed using the miRTarBase and TRRUST databases (**Figure 11**). Network analysis predicted multiple miRNAs and TFs as potential upstream regulators. Notably, CEBPG, FOXA1, NFKB1, KLF6, and SP1 were identified as highly interconnected factors within this regulatory network, each targeting distinct combinations of the key genes. Given that transcription factors such as NFKB1 are classical mediators of inflammatory signaling cascades, their emergence as central nodes suggests a potential transcriptional link between mucosal inflammation and stromal cell activation.

### 3.9 Confirmation of key gene expression levels in the experimental colitis

**Figure 12:**
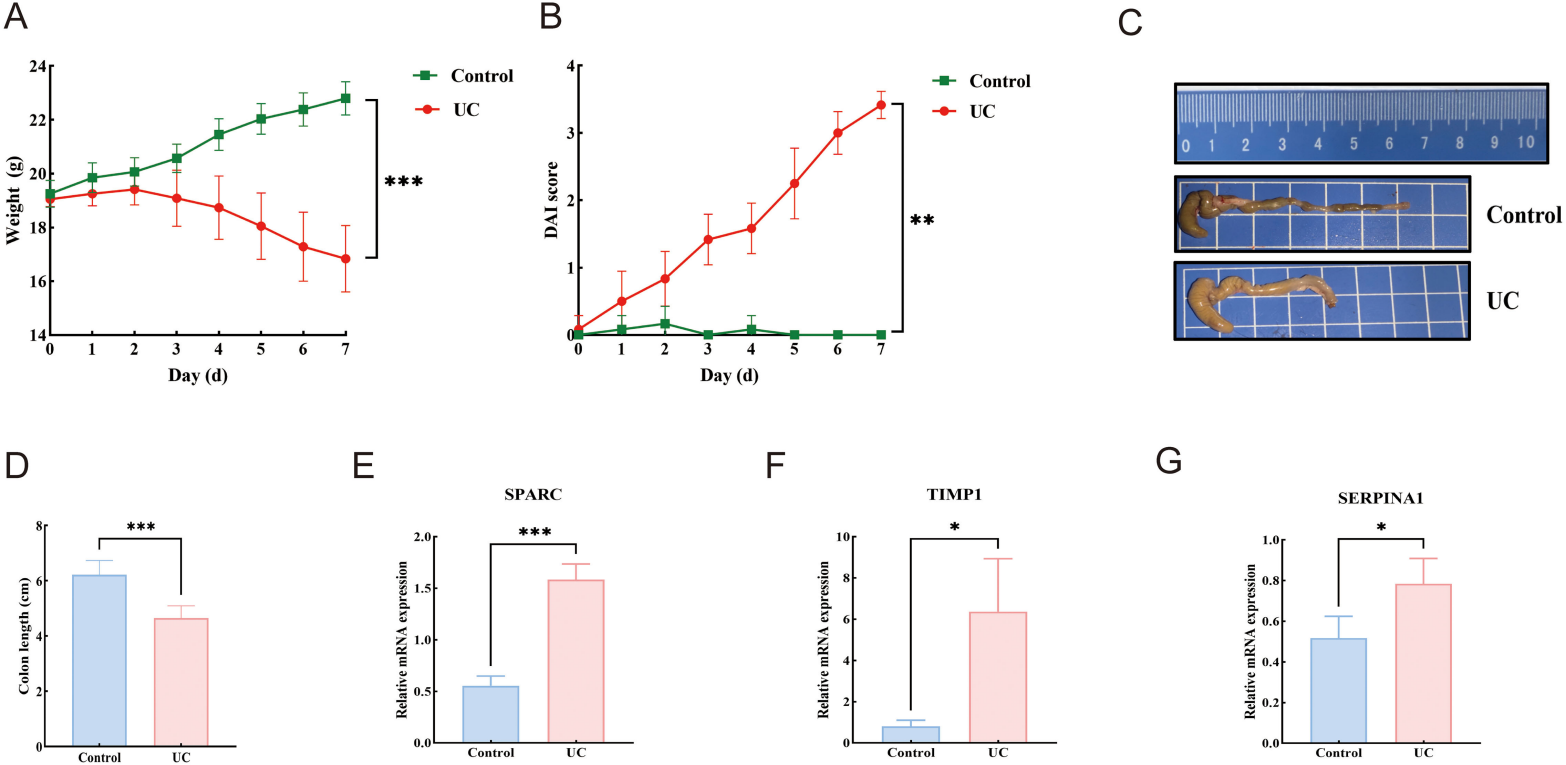
Validation of key genes in DSS-induced UC mouse model. **(A)** Body weight after DSS treatment (*n* = 6). **(B)** DAI score after DSS treatment (*n* = 6). **(C)** Representative intestinal tissue image of mice (*n* = 6). **(D)** Colon length of mice (*n* = 6). **(E)** The mRNA expression of SPARC (*n* = 3). **(F)** The mRNA expression of TIMP1 (*n* = 3). **(G)** The mRNA expression of SERPINA1 (*n* = 3). Data are presented as mean ± SD (error bars). *P < 0.05, **P < 0.01, ***P < 0.001 vs. the control group.

**Figure 13:**
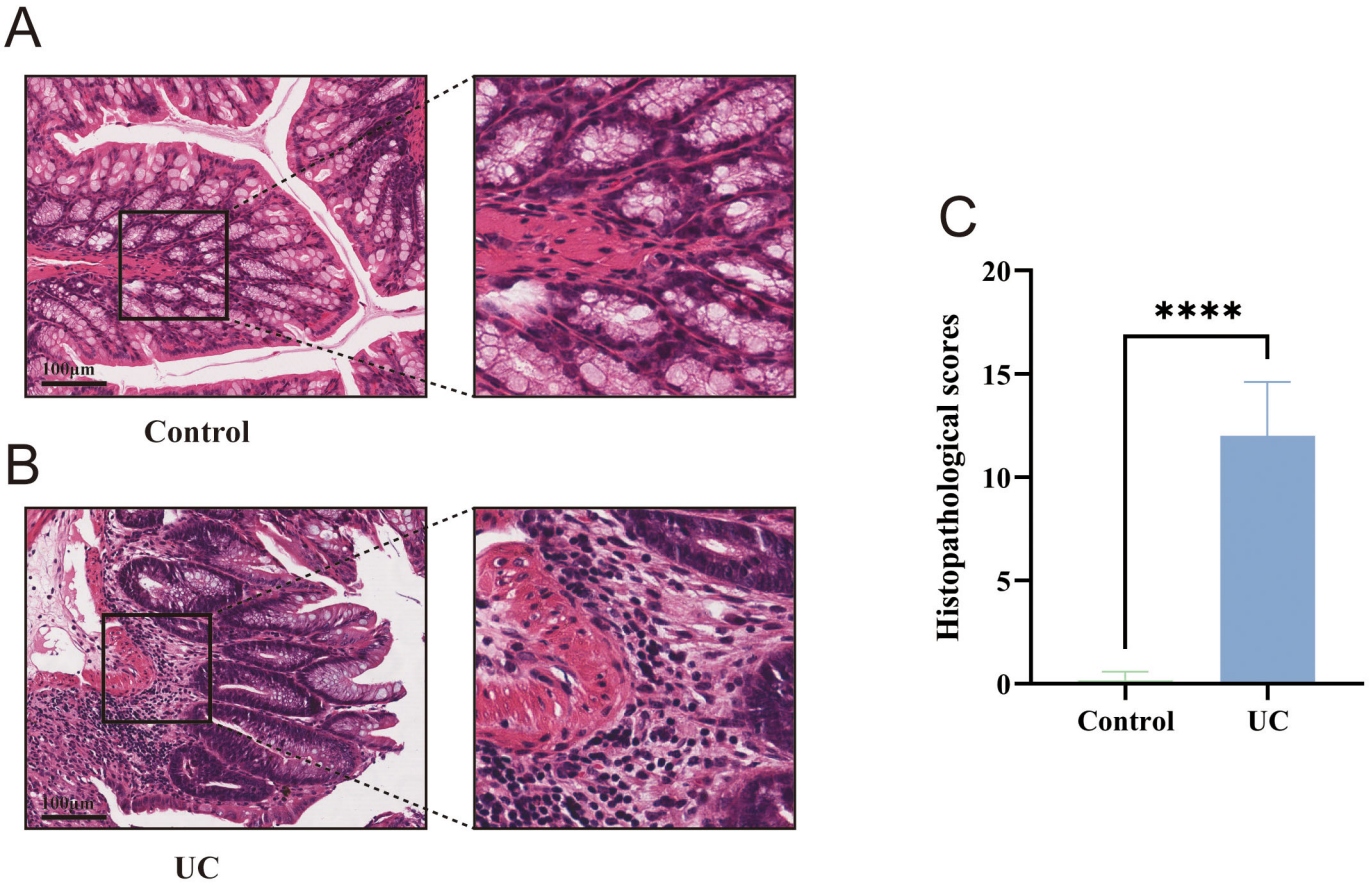
HE staining to observe the damage of colonic tissue in mice. **(A)** The control group of HE staining (*n* = 6). **(B)** The UC model group of HE staining (*n* = 6). **(C)** Difference of histopathological scores between the control and the UC group (*n* = 6). Data are presented as mean ± SD (error bars). ****P < 0.0001 vs. the control group.

To confirm the bioinformatics findings, mouse models of UC were established by administering 3% DSS for 7 days. Each group consisted of six mice. Compared with the control group, mice in the UC group showed a significant time-dependent reduction in body weight (**Figure 12A**), accompanied by consistently elevated DAI scores (**Figure 12B**). On day 8, the entire colon was excised for observation and length measurement. The intestinal mucosa in the UC group exhibited erosion, congestion, and edema (**Figure 12C**). Further assessment revealed that colon length in the UC group (4.65 ± 0.45 cm) was significantly shorter than that in the control group (6.22 ± 0.52 cm) (*P* < 0.001) (**Figure 12D**). For histopathological validation, distal colon tissues were subjected to H&E staining. The UC group exhibited typical pathological features of UC, including impaired epithelial structure, mucosal injury and erosion, damaged intrinsic glands, distorted or lost crypt architecture, blurred or absent glandular structures, and extensive infiltration of inflammatory cells (**Figures 13A, B**). The histopathological score was significantly higher in the UC group than in the control group (*P* < 0.001) (**Figure 13C**). These changes intuitively indicated the development of UC in mice, demonstrating that the UC model was successfully constructed. The expression levels of the three key genes were determined by qRT-PCR (three biological replicates). The results showed that the mRNA levels of SPARC (*P* < 0.001), TIMP1 (*P* = 0.023), and SERPINA1 (*P* = 0.048) were significantly higher in the UC group than in the control group, which needs further mechanistic evidence (**Figures 12E–G**).

## 4 Discussion

UC is a chronic and refractory inflammatory bowel disease characterized by mucosal erosion, persistent inflammation, and progressive fibrosis (13). Despite its increasing global incidence and substantial socioeconomic burden, current therapies still fail to induce durable remission in a considerable proportion of patients, largely due to an incomplete understanding of the complex interactions within the intestinal microenvironment. Distinct from the traditional view of platelets as merely hemostatic factors, emerging evidence recognizes them as key immune modulators that actively shape the inflammatory landscape (14–17). Platelet activation plays a critical role in UC progression, serving as an important bridge connecting vascular injury with immune–stromal dysfunction. In the UC colon, injured endothelial cells and microbial translocation trigger aberrant platelet activation, prompting the degranulation of alpha and dense granules (18, 19). This process releases a large array of pro-inflammatory and pro-fibrotic mediators into the local mucosa. Critically, Guy et al. demonstrated that activated platelets recruit leukocytes through P-selectin-PSGL-1, amplifying oxidative stress and neutrophil extracellular trap formation (NETosis) (20), whereas Li et al. found that platelet-derived signals activate macrophages and dendritic cells (21). Moreover, accumulating evidence indicates that platelet-derived signals (e.g., TGF-β and PDGF) can permeate the tissue matrix, acting as potent upstream stimuli that reprogram local fibroblasts and drive ECM remodeling (22–24). Therefore, a systematic investigation of transcriptomic biomarkers associated with platelet activation is essential not only for assessing mucosal tissue remodeling but also for providing a foundation to develop novel, microenvironment-targeted therapeutic strategies, ultimately opening new avenues to improve disease management and patient outcomes.

In this study, we employed an integrative bioinformatics approach to elucidate transcriptomic signatures of platelet activation in UC. We identified 22 overlapping genes between UC and platelet activation, which were highly enriched in platelet degranulation, ECM organization, and chemokine signaling. Using three machine learning algorithms, we identified three key platelet activation-related genes (PARGs): SPARC, TIMP1, and SERPINA1. ROC analysis confirmed their robust diagnostic performance in both the training cohort and three independent validation cohorts. At the functional level, GSEA revealed that upregulation of these genes is associated with ECM receptor interactions and pro-inflammatory pathways, whereas their downregulation aligns with colonic metabolic homeostasis (e.g., butyrate metabolism). Furthermore, our regulatory network predicted that classical inflammatory mediators (e.g., NFKB1 and SP1) serve as key upstream transcriptional regulators. Single-cell analysis localized these genes predominantly to intestinal fibroblasts. Finally, we validated these bioinformatics findings in a DSS-induced colitis mouse model, which successfully recapitulated classic UC characteristics (weight loss, elevated DAI, and mucosal injury), and confirmed significant upregulation of these genes *in vivo* by qRT-PCR. Taken together, these findings suggest that these fibroblast-localized PARGs may be involved in the pathological progression of UC.

Our immune infiltration analysis revealed significant remodeling of the intestinal immune microenvironment in UC. These changes may exacerbate inflammatory cell infiltration and fibrosis processes (7, 25–27). Emerging evidence suggests that high enrichment of activated memory CD4^+^ T cells promotes Th1/Th17 polarization, induces epithelial cell apoptosis, and disrupts tight junctions (e.g., occludin and claudin), thereby damaging the intestinal barrier (28–30). Moreover, increased M1 macrophages drive the production of TNF-α, IL-1β, IL-6, and reactive oxygen species (ROS), which directly damage the intestinal epithelium and amplify the inflammatory cascade through the NF-κB pathway (31–34). Meanwhile, neutrophil infiltration leads to the release of myeloperoxidase (MPO) and neutrophil extracellular traps (NETs), which severely degrade the epithelial basement membrane and induce crypt necrosis (35). Additionally, activated mast cells release histamine and proteases, increasing vascular permeability and exacerbating mucosal edema (36–38). Collectively, these findings indicated that the three key genes may be associated with ECM homeostasis and immune polarization, thereby contributing to UC progression.

Secreted protein acidic and rich in cysteine (SPARC) is a matricellular protein that plays a critical role in cell adhesion, ECM production, and cell cycle regulation, and has been demonstrated to drive the progression of inflammatory bowel disease (IBD) (39, 40). Camarillo et al. reported that SPARC mRNA levels are elevated in UC patients and correlate with histological activity, and deeply, Tanaka et al. demonstrated that SPARC knockout alleviates colitis by modulating Th17 cell differentiation (40, 41). Moreover, SPARC has been shown to promote macrophage polarization toward a pro-inflammatory phenotype (39). Similarly, tissue inhibitor of metalloproteinase 1 (TIMP1), initially identified as an endogenous inhibitor of matrix metalloproteinases (MMPs), has recently been recognized as a pleiotropic cytokine that promotes cell survival, proliferation, and inflammatory responses (42). Elevated TIMP1 levels are associated with poor prognosis in various diseases, including cancers, pancreatitis, and viral infections (43–46). In the context of IBD, studies by Louis et al. and Arihiro et al. have confirmed increased TIMP1 expression in both mucosal and plasma samples, which positively correlates with endoscopic disease severity and C-reactive protein levels (47, 48). Subsequently, Minh et al. identified TIMP1 as a major regulator of ECM remodeling in IBD (49). Although the upstream triggers driving the concurrent accumulation of these two proteins in the UC stroma remain incompletely understood, our findings provide a potential explanation. Following platelet activation, alpha granule degranulation triggers the abundant release of growth factors such as TGF-β and PDGF. According to our regulatory network, these factors may activate downstream transcription factors (e.g., SP1 and SMAD), which in turn stimulate intestinal fibroblasts. We hypothesize that this platelet-induced, fibroblast-derived synergistic upregulation of SPARC and TIMP1 may contribute to the inflammation–fibrosis progression in UC.

In contrast to the potential pro-fibrotic roles of SPARC and TIMP1, the microenvironmental remodeling in UC also exhibits features of compensatory adaptation, best exemplified by SERPINA1. This gene encodes alpha-1-antitrypsin (AAT), a classic serine protease inhibitor that maintains mucosal barrier integrity by neutralizing neutrophil elastase and preventing degradation of epithelial tight junctions (50–53). As UC progresses, massive neutrophil infiltration inevitably releases destructive proteases. Although AAT is traditionally synthesized by hepatocytes and stored in small amounts within platelet alpha granules, our single-cell analysis revealed its abundant accumulation in local fibroblasts of the UC mucosa, suggesting that activated fibroblasts may attempt to neutralize the neutrophil-driven “protease storm” to protect residual epithelial integrity. Thus, the activation of fibroblasts by upstream platelet signals does not appear to be a unidirectional pathological cascade but rather involves a dynamic balance between ECM deposition and compensatory barrier defense.

Several limitations of this study must be acknowledged. First, reliance on public databases inevitably introduces dataset heterogeneity and cross-platform effects. Moreover, our validation cohorts are relatively small; therefore, large-scale, multicenter prospective clinical studies are warranted to further validate the diagnostic robustness of these markers. Second, algorithms such as CIBERSORT and GSEA depend on predefined gene signatures to estimate cell abundance and pathway enrichment, which may be prone to overfitting and cannot fully capture dynamic cellular states *in vivo*. Third, this study primarily relies on correlative transcriptomic data and limited qRT-PCR validation in a DSS-induced mouse model. The current findings represent transcriptional associations rather than definitive mechanistic evidence. Extensive functional validation, including gene knockout/overexpression animal models, *in vitro* co-culture systems, and spatial transcriptomics, is essential to determine whether platelet activation causally drives fibroblast reprogramming and to functionally dissect the impact of SPARC, TIMP1, and SERPINA1 on stromal–immune dysfunction during UC progression.

## 5 Conclusion

In conclusion, this study integrated WGCNA and machine learning algorithms to identify three key PARGs (SPARC, TIMP1, and SERPINA1) in UC, and systematically characterized their diagnostic value and biological relevance. These findings provide a new perspective on the complex pathogenesis of UC and lay a foundation for future therapeutic development. Crucially, these candidate platelet activation-related gene associations require further mechanistic validation.

## Data Availability

The original contributions presented in the study are included in the article/supplementary material. Further inquiries can be directed to the corresponding authors.
